# Evaluating the efficacy of vascularized pedicled skin flaps in treating sacrococcygeal pressure ulcers

**DOI:** 10.1016/j.jpra.2025.06.014

**Published:** 2025-06-28

**Authors:** Tan Anh Tran, Khanh Trinh Quoc Pham, Dat Ke Vo, Nhan Hoang Nguyen, Nhan Hong Nguyen, Phi Duong Nguyen

**Affiliations:** a Trung Vuong Hospital, Ho Chi Minh City, Vietnam; b Tra Vinh University, Tra Vinh, Vietnam; c City Children’s Hospital, Ho Chi Minh City, Vietnam; d Can Tho University of Medicine and Pharmacy, Can Tho City, Vietnam

**Keywords:** Sacrococcygeal pressure ulcers, Vascularized pedicled skin flaps, Surgical outcomes, Wound healing, Postoperative care

## Abstract

**Background:**

Pressure ulcers, particularly sacrococcygeal ulcers, represent a persistent healthcare challenge despite being preventable. This study aims to evaluate the outcomes of using vascularized pedicled skin flaps for sacrococcygeal pressure ulcer treatment and to identify factors influencing these outcomes at our hospital.

**Methods:**

A descriptive case series study was conducted on 42 patients with sacrococcygeal pressure ulcers treated using pedicled skin flaps with preserved vascular supply from May 2018 to May 2023.

**Results:**

The study population had a female-to-male ratio of 6:4, with a mean age of 72.5 years (range: 27–97 years). A majority (61.9 %) of patients received weight-bearing flaps. Favorable outcomes were observed in 34 cases (80.9 %) at 2 weeks and in 35 cases (83.3 %) at 1 month post-surgery. Key factors influencing outcomes included ulcer size, flap dimensions, and the duration of postoperative hospitalization.

**Conclusion:**

Pedicled flap reconstruction remains effective for sacrococcygeal pressure ulcer management. Flap selection should be individualized, taking into account ulcer size, patient mobility, and surgical goals. While outcomes varied across flap types, this study supports a tailored, experience-based approach rather than asserting one flap's superiority. Further comparative research is warranted.

## Introduction

Pressure ulcers, particularly those in individuals requiring prolonged hospitalization, are prevalent in the sacrococcygeal region, accounting for approximately one-quarter of all ulcer locations.^1^ Data from studies conducted in the United States reveal that 32–40 % of patients with spinal cord injuries develop ulcers during their initial hospital stay, while 26–31 % of intensive care unit patients experience sacrococcygeal pressure ulcers.^2^^,^^3^ These ulcers significantly impact treatment outcomes, prolong hospitalization, diminish patients’ quality of life, and elevate healthcare costs.

Effective management of pressure ulcers necessitates a multidisciplinary approach that integrates internal medicine, nutritional support, wound care, physical therapy, and surgical intervention.^4^ Among surgical techniques, the use of pedicled flaps with preserved vascular supply, including perforator (fasciocutaneous) flaps and musculocutaneous flaps, has gained prominence. However, there remains limited guidance on selecting the optimal flap type for individual patient characteristics, particularly in cases with heterogeneous comorbidities.

This study, conducted at the Burn, Plastic, and Aesthetic Department of our hospital, aimed to evaluate the outcomes of vascularized pedicled skin flap transfer methods in treating sacrococcygeal pressure ulcers. The research objectives included assessing the effectiveness of these techniques and identifying factors influencing treatment outcomes. The findings are intended to provide valuable insights for selecting suitable flap types and improving surgical strategies for managing sacrococcygeal pressure ulcers.

## Materials and methods

This was a consecutive case series study conducted between May 2018 and May 2023. A total of 47 patients were initially evaluated. Five patients were excluded: two patients died before surgical intervention, one was transferred to another facility for unrelated comorbidities, and two were lost to follow-up after discharge. Thus, 42 patients were included in the final analysis.

The inclusion criteria were as follows: (1) patients treated in the Burn, Plastic, and Aesthetic Department of our hospital with a clinical diagnosis of stage III or IV sacrococcygeal pressure ulcers; (2) patients who underwent surgical intervention using vascularized pedicled skin flaps; (3) patients meeting the following laboratory parameters to ensure adequate postoperative wound healing: protein/blood ≥ 2 g/dL, albumin/globulin ratio ≥ 1/2, hemoglobin ≥ 10 g/dL, and hematocrit ≥ 30 %; and (4) patients who provided informed consent to participate in the study.

Exclusion criteria included: (1) patients who did not attend follow-up visits or were lost to follow-up after discharge; (2) patients deemed unfit for surgery (classified as ASA grades 4, 5, or 6 by the American Society of Anesthesiologists); (3) patients who died or were transferred during the study period; and (4) cases with incomplete or insufficient follow-up data, defined as missing >20 % of the required information.

Ulcer size (length and width) was measured preoperatively using sterile flexible rulers. The longest dimension (head-to-toe) and the widest perpendicular dimension (side-to-side) were recorded, and area was estimated by multiplying length and width, consistent with standard wound measurement guidelines. The association between ulcer size (length, width, area) and complication rates was analyzed using univariate analysis. Chi-square tests were used for categorical variables, and logistic regression was employed to evaluate the predictive value of ulcer dimensions on complication risk. A *p*-value <0.05 was considered statistically significant.

Wound healing time was defined as the duration from surgery to complete epithelialization and stable wound closure without drainage. Sutures were typically removed between postoperative day 12 and day 16 depending on individual wound status. Healing was assessed clinically at 2 weeks and at 1 month.

In cases with malodorous purulent discharge, aggressive debridement and wound bed preparation were performed prior to definitive flap coverage. In patients with controlled infection after debridement and antibiotics, a one-stage flap reconstruction was undertaken. In cases of persistent infection, staged procedures were considered but were infrequent in our series.

After surgery, patients remained on bed rest for a minimum of 14 days. Patients were positioned prone or laterally, avoiding direct pressure on the reconstructed area. Low air-loss pressure-relieving mattresses were routinely used. Patient repositioning was performed every 2 hours. Dressing protocols included a non-adherent silicone layer (e.g., Mepitel) covered with sterile gauze and absorbent secondary dressings. Dressings were changed every 48–72 h unless wound drainage required more frequent changes.

## Results

The study included 42 patients, of which 25 (59.5 %) were female and 17 (40.5 %) were male. The median age of participants was 72.5 years, with a range of 27–97 years. The study population included a wide range of patients, from 27 to 97 years old, encompassing both geriatric patients with debility and younger patients with spinal cord injuries. A significant proportion of patients (97.6 %) presented with comorbidities. The primary causes of secondary ulcers were stroke or cerebral palsy (33.3 %) and debilitation from other conditions (31.0 %), while paralysis due to spinal injury accounted for 7.1 % of cases (Table 1).

**Table 1:** General characteristics of study subjects (*n* = 42).

Characteristic		Frequency (n)	Ratio (%)
Gender	Male	17	40.5
	Female	25	59.5
Age	Median (IQR)	72.5 (64–80)	
	Range (Min-Max)	27–97	
Causes of secondary ulcers	Paralysis due to spinal injury	3	7.1
	Stroke/Cerebral palsy	14	33.3
	Hip fracture	1	2.4
	Debilitation from other causes	13	31.0
	Other causes	14	33.3
Comorbidities	Yes	41	97.6
	No	1	2.1
Total		42	100.0

The majority of patients (83.3 %) had stage IV sacrococcygeal pressure ulcers, with the remaining 16.7 % diagnosed at stage III. The median ulcer area was 63 cm², with a range of 20 to 336 cm². Malodorous purulent discharge was observed in 79 % of cases, and edema was noted in 76 % (Table 2).

**Table 2:** Clinical characteristics of ulcers (*n* = 42).

Characteristic		Frequency (n)	Ratio (%)
Ulcer stage	Stage III	7	16.7
	Stage IV	35	83.3
Ulcer length	Mean ± SD	9.3 ± 3.5	
	Range (Min-Max)	5–21	
Ulcer width	Mean ± SD	8.0 ± 2.8	
	Range (Min-Max)	4–16	
Ulcer area	Median (IQR)	63 (40–121)	
	Range (Min-Max)	20–336	
Ulcer condition	Edema	32	76.0
	Granulation tissue	11	26.0
	Malodorous purulent discharge	33	79.0
	Sinus tracts	15	36.0
Total		42	100.0

**Figure 1:**
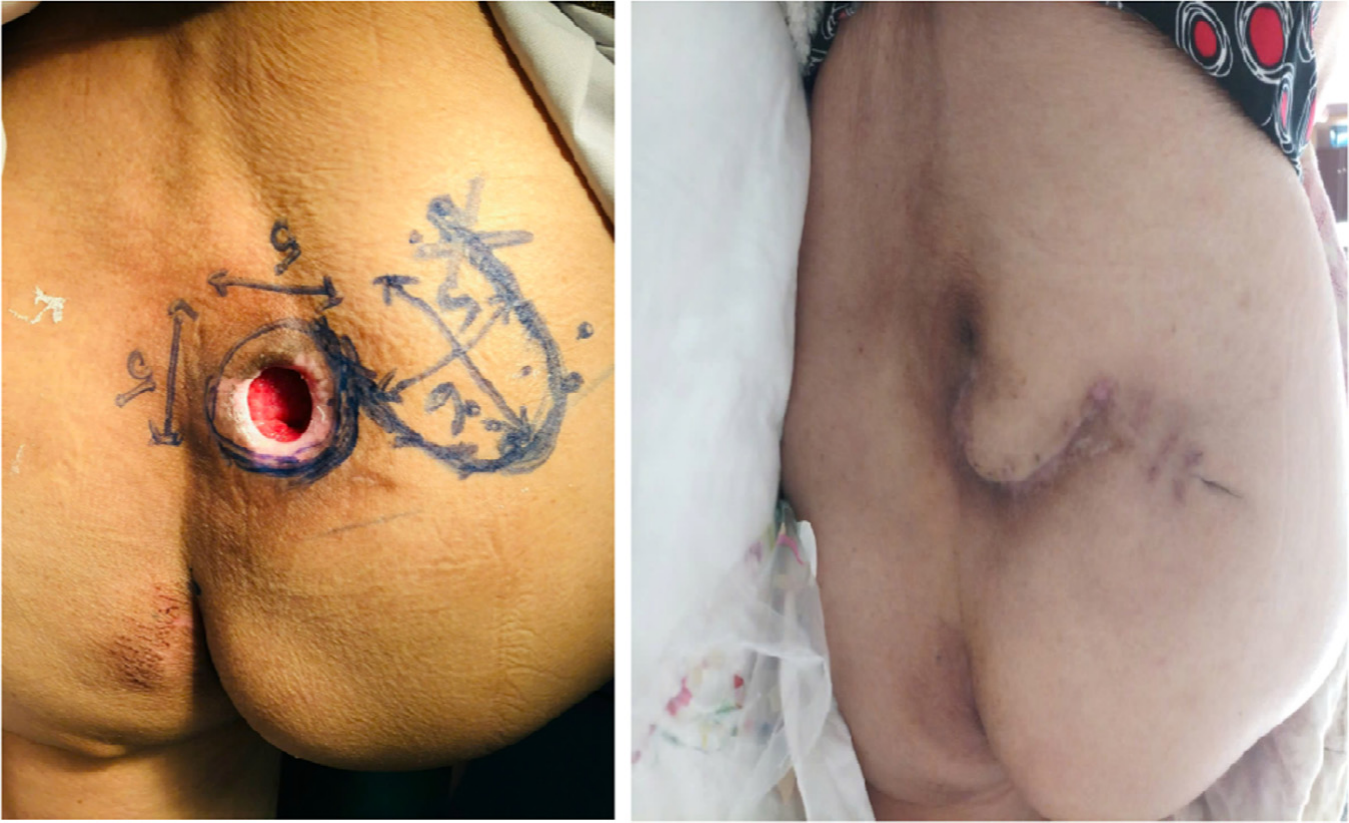
Fasciocutaneous flap pre-operative and postoperative.

**Figure 2:**
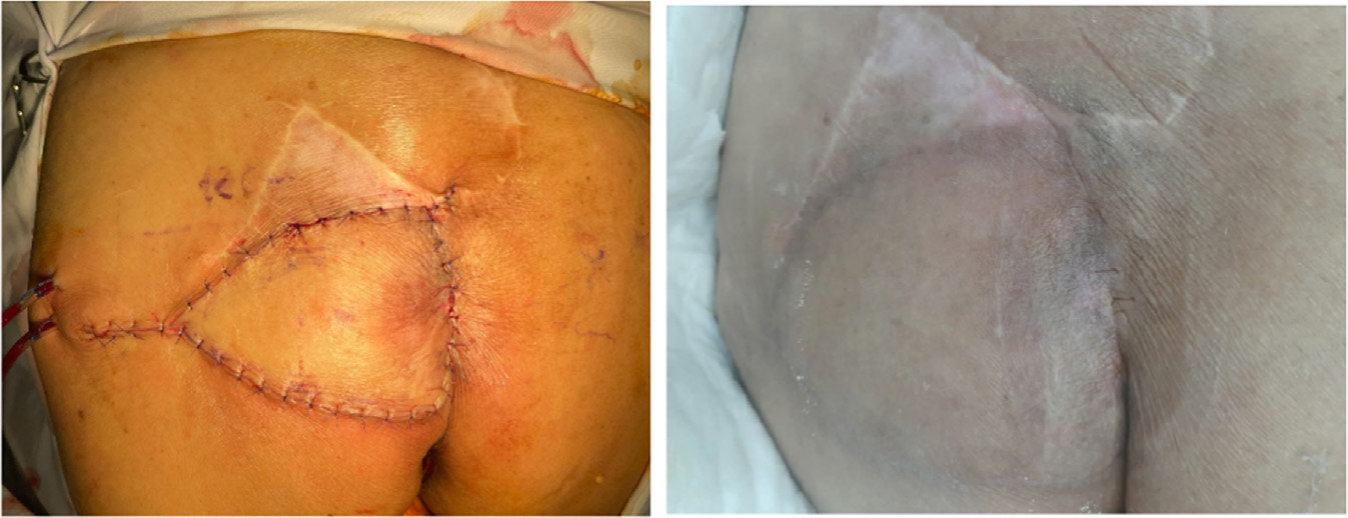
Perforator flap postoperative and 1 month postoperative.

**Figure 3:**
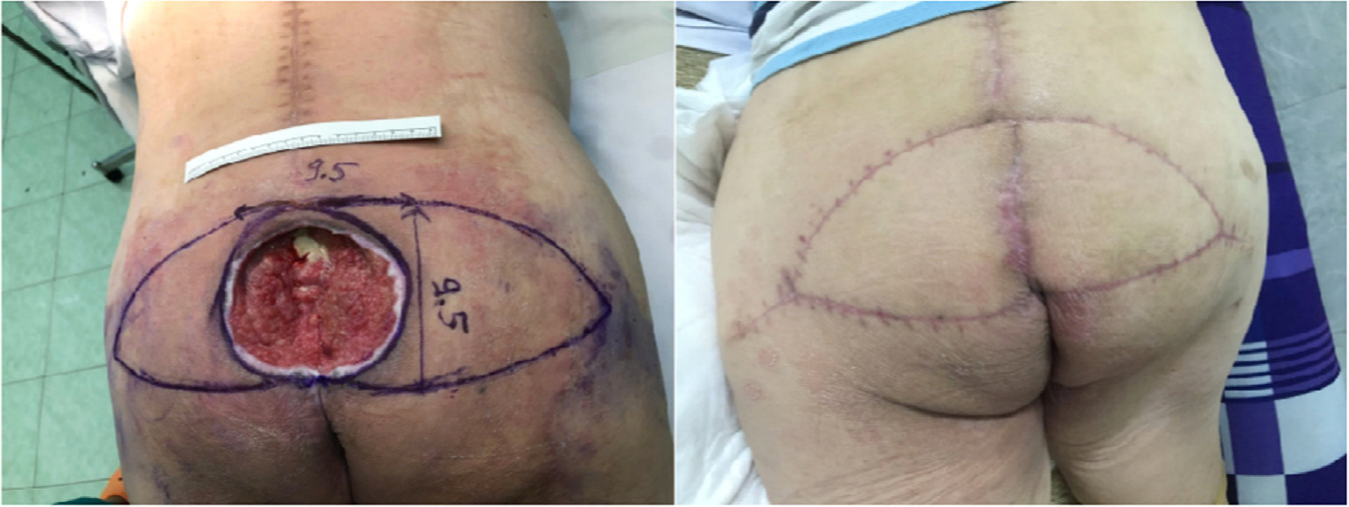
Gluteus maximus muscle flap pre-operative and postoperative.

The fasciocutaneous flap (Figure 1) was the most frequently used technique (61.9 %), followed by perforator flaps (Figure 2) (23.8 %) and gluteus maximus muscle flaps (Figure 3) (14.3 %). The mean wound healing time was 15.3 days, with a range of 7 to 25 days. After 2 weeks, 80.9 % of patients showed favorable outcomes, increasing to 83.3 % after 1 month (Table 3).

**Table 3:** Characteristics of skin flap applications (*n* = 42).

Characteristic		Frequency (n)	Ratio (%)
Type of flap used	Fasciocutaneous flap	26	61.9
	Gluteus maximus muscle flap	6	14.3
	Perforator flap	10	23.8
Flap length	Mean ± SD	10.4 ± 3.4	
	Range (Min-Max)	6–22	
Flap width	Mean ± SD	8.0 ± 3.0	
	Range (Min-Max)	4–16	
Ulcer area	Median (IQR)	84 (45–132)	
	Range (Min-Max)	24–352	
Surgical duration	Median (IQR)	95 (90–120)	
	Range (Min-Max)	45–210	
Wound healing time	Mean ± SD	15.3 ± 4.7	
	Range (Min-Max)	7–25	
Post-surgical hospital stay	Median (IQR)	19.5 (14–24)	
	Range (Min-Max)	7–63	
Total		42	100.0

Intraoperative complications were observed in 4 patients (9.6 %), including minor bleeding requiring additional hemostasis (2 cases), flap torsion (1 case), and transient pedicle compression (1 case), all of which were corrected during surgery without impacting final flap viability.

After 2 weeks, 34 cases (80.9 %) showed good outcomes, while 1 month later, the number increased to 35 (83.3 %) (Table 4).

**Table 4:** Evaluation of outcomes of skin flap applications (*n* = 42).

Characteristic		Frequency (n)	Ratio (%)
Outcomes after 2 weeks	Good	34	80.9
	Fair	7	16.7
	Poor	1	2.4
Outcomes after 1 month	Good	35	83.3
	Fair	6	14.3
	Poor	1	2.4
Total		42	100.0

At the 2-week follow-up, the overall rate of patients without complications was 90.4 %. Fasciocutaneous flaps accounted for 65.8 % of these cases, gluteus maximus muscle flaps 13.2 %, and perforator flaps 21 %. The remaining cases (9.6 %) experienced complications, including bleeding (2.4 %), flap necrosis (2.4 %), seroma post-drain removal (2.4 %), and other complications (2.4 %). No cases of pedicle compression were observed (Table 5).

**Table 5:** Complications after 2 weeks (*n* = 42).

Complication	Fasciocutaneous flap		Gluteus maximus muscle flap		Perforator flap		Total	
	N	%	N	%	N	%	N	%
No complications	25	65.8	5	13.2	8	21.0	38	90.4
Bleeding	0	0	0	0	1	100	1	2.4
Flap necrosis	0	0	0	0	1	100	1	2.4
Pedicle compression	0	0	0	0	0	0	0	0
Seroma Post-drain removal	1	100	0	0	0	0	1	2.4
Others	0	0	1	100	0	0	1	2.4

At 1 month postoperatively, 35 patients (83.3 %) achieved good wound healing outcomes as defined by stable wound closure without drainage. Eight cases (19.0 %) exhibited delayed healing or partial wound dehiscence. However, not all patients achieving wound closure were fully mobilized at 1 month, particularly among elderly and spinal cord injury patients with significant comorbidities. Mobilization timing varied and was not synonymous with wound closure.

At the 1-month follow-up, complications were reported in 10 cases (23.8 %). Flap edge necrosis was the most common complication, observed in six cases (60 %). Necrosis affecting more than one-third of the flap occurred in one case (10 %). Other complications, which included two cases of epidermolysis and one case of incomplete debridement of necrotizing sacrococcygeal osteomyelitis, were reported in three cases (30 %) (Table 6).

**Table 6:** Complications after 1 month (*n* = 42).

Complication		Fasciocutaneous flap	Gluteus maximus muscle flap	Perforator flap	Total
Flap edge necrosis	Frequency	4	1	1	6
	Ratio (%)	66.6	16.7	16.7	60.0
Necrosis >1/3 of flap	Frequency	0	0	1	1
	Ratio (%)	0	0	0	10.0
Other complications	Frequency	2	1	0	3
	Ratio (%)	66.7	33.3	0	30.0

The analysis revealed that ulcer size, flap size, and post-surgical hospital stay significantly influenced treatment outcomes. Larger ulcers, particularly in terms of length and width, were associated with less favorable outcomes after 2 weeks (*p* = 0.042 and 0.016, respectively) and 1 month (*p* = 0.054 and 0.022, respectively). Similarly, larger flap dimensions, including length and width, were associated with higher complication rates (*p* = 0.043 and 0.012, respectively). The duration of post-surgical hospital stay also had a significant association with outcomes. Longer hospital stays were correlated with poor treatment results after 1 month (*p* = 0.018) and increased complication rates (*p* = 0.006). Surgical duration, however, showed no significant association with treatment outcomes. These findings emphasize the importance of optimizing flap selection and surgical planning to improve outcomes in patients with sacrococcygeal pressure ulcers (Table 7).

**Table 7:** Factors associated with the treatment outcomes of sacrococcygeal pressure ulcers using vascularized pedicled skin flaps (*n* = 42).

Related factors	Treatment outcome after 2 weeks (*p*-value)	Treatment outcome after 1 month (*p*-value)	Complications (*p*-value)
Ulcer size Length Width Area	0.042 0.016 0.053	0.054 0.022 0.064	0.084 0.033 0.100
Flap size Length Width Area	0.043 0.012 0.308	0.056 0.019 0.067	0.089 0.015 0.076
Duration Surgical duration Post-surgery hospital stay	0.308 0.004	0.247 0.018	0.546 0.006

## Discussion

The study subjects had a median age of 72.5 years, which aligns with the epidemiology of pressure ulcers observed both in Vietnam and globally.^1^^,^^3^ The male-to-female ratio in this study was approximately balanced. Pressure ulcers are prevalent among individuals at high risk, often presenting with multiple comorbid conditions.^5^ In our cohort of 42 patients, 97.6 % had associated comorbidities, emphasizing the critical need for managing underlying health issues in treating pressure ulcers. Early postoperative care, including strict bed rest with pressure-relieving mattresses and scheduled repositioning, contributed significantly to protecting flap viability and optimizing outcomes. Mobilization protocols were tailored to wound status and overall patient condition.

Secondary causes of pressure ulcers frequently stem from conditions requiring prolonged immobilization, such as spinal cord injuries, stroke, burns, or other chronic illnesses.^6^ Among our patients, the duration of ulcer presence before admission ranged from 1 to 3 months, which explains the high proportion of advanced-stage ulcers due to prolonged tissue damage. The ulcers had a median area of 63 cm², ranging from 20 to 336 cm². These dimensions, comparable to findings in global and Vietnamese studies, highlight the challenges posed by large ulcers, including compromised blood supply and heightened infection risks.^7^ As expected, patients with spinal cord injuries (SCI) and elderly patients with multiple comorbidities demonstrated a tendency toward slower wound healing. However, with meticulous surgical technique, nutritional optimization, and intensive wound care, mean healing time for the cohort remained approximately 2 weeks. This timeframe corresponds to clinical wound stability suitable for suture removal and gradual mobilization but does not necessarily reflect complete tissue remodeling.

The clinical characteristics of ulcers observed included widespread edema, tissue necrosis, osteomyelitis, and malodorous exudate, with the latter being the most common feature (79 %). Similar trends have been documented in other Vietnamese studies.^7^ Conservative treatments are insufficient for stage III and IV ulcers, necessitating surgical interventions. Fasciocutaneous flaps were the most frequently used method in our study, followed by perforator flaps and gluteus maximus muscle flaps, corroborating findings from prior Vietnamese research on flap outcomes.^7^^,^^8^

The median flap size in our study was 84 cm², larger than those reported in international studies.^9^, ^10^, ^11^ This discrepancy may reflect differences in ulcer characteristics and surgical expertise. Surgical duration, recorded at a median of 95 min, varied by flap type. Extended surgical times were associated with increased risks, including blood loss, delayed wound healing, and higher inflammatory responses. Despite these challenges, the mean wound healing time in our study was 15 days, consistent with prior Vietnamese research.^10^ However, eight cases failed to achieve wound closure within 30 days, underscoring the importance of timely wound care and targeted antibiotic therapy.

The mean hospital stay in this study was longer than the 26 days reported by Khurram et al.,^9^ attributed to the advanced ulcer stages and patient comorbidities. Recovery time depended significantly on the duration of ulcer presence prior to admission, with delayed care exacerbating outcomes. After 2 weeks, 80.9 % of cases showed good outcomes, increasing to 83.3 % after 1 month. Early-stage postoperative results aligned with Phan Nhat Khanh et al., who reported 87.5 % good outcomes in a similar patient group.^12^

The complication rate during surgery was 9.6 %, encompassing issues such as bleeding, flap torsion, and seroma formation. Suture line rupture was a notable complication, consistent with findings from Schryvers et al.^13^ Complications post-surgery were managed proactively, minimizing the impact on recovery. Proactive management of wound healing complications involved early identification of wound problems, prompt bedside interventions such as partial suture removal for drainage, minor debridement of nonviable tissue, local wound care optimization, timely use of targeted antibiotics based on wound cultures, and, if necessary, minor surgical revision. This approach minimized progression of minor complications into major flap failure.

Effective treatment of sacrococcygeal pressure ulcers demands comprehensive care spanning preoperative preparation, surgical intervention, and postoperative follow-up. Among the 10 complications observed 1 month post-surgery, flap edge necrosis (60 %) was predominant. These findings are comparable to those from Chinese studies reporting partial dehiscence and necrosis rates of 20.3 % and flap failure rates of 7.2 %.^11^

Our results revealed that larger ulcer sizes negatively impacted treatment outcomes and increased complication rates, corroborating prior studies indicating that extensive wounds require longer recovery periods.^14^ Additionally, a strong relationship was observed between flap size and outcomes, with larger flaps associated with poorer results. This contrasts with Foster et al., who argued that flap size does not significantly affect surgical success but rather depends on flap type.^15^

The duration of hospital stay was a key determinant of treatment outcomes. Longer stays were associated with increased complication rates, aligning with findings by Jaul that efficient care leads to better results.^3^ Conversely, Sorensen emphasized the risks of insufficient postoperative care, highlighting the need for balance.^14^ Extended hospitalization can increase the likelihood of hospital-acquired infections, further complicating recovery and emphasizing the need for efficient yet high-quality care delivery systems.

Our study included a heterogeneous patient population, which reflects the real-world complexity of sacrococcygeal pressure ulcer management. This heterogeneity, including differences in age and comorbidities, may influence outcomes and limits the generalizability of our findings. Future stratified analyses may be warranted to refine flap selection criteria.

The clinical outcomes observed in this study suggest favorable healing with various pedicled skin flap techniques. However, an important limitation is that flap types were not randomly assigned but rather selected based on ulcer size, location, and patient condition. Specifically, musculocutaneous flaps, particularly the gluteus maximus flap, were reserved for larger, more complex defects and for patients with profound immobility. Therefore, the increased complication rate associated with these flaps may reflect the underlying complexity of the clinical situation rather than an intrinsic disadvantage of the flap type itself.

As such, while our data demonstrate outcome trends across different flap types, they do not support a direct comparison of flap efficacy for similar indications. The study design does not allow us to assert the superiority of one flap type over another. Instead, we propose a pragmatic algorithm for flap selection based on patient-specific factors, including ulcer size, mobility status, and comorbidities.

Additionally, the concern regarding the potential for increased local pressure and risk of ulcer recurrence due to the bulk of full-thickness gluteus maximus musculocutaneous flaps is acknowledged. To mitigate this risk, careful intraoperative sculpting of the flap and meticulous postoperative positioning are essential. Pressure-relieving strategies should be strictly enforced to prevent recurrence, especially in high-risk patients.

In light of these factors, our recommendations serve more as guidance derived from clinical practice rather than evidence of differential flap superiority.

## Conclusion

In conclusion, vascularized pedicled skin flaps offer a reliable reconstructive option for sacrococcygeal pressure ulcers when tailored to patient-specific characteristics. While fasciocutaneous and perforator flaps were predominantly used for smaller to moderate ulcers in patients with partial mobility, musculocutaneous flaps were selected for extensive defects in immobile patients. The study’s findings do not establish the superiority of any single flap type due to the lack of direct comparisons across uniform clinical scenarios. Rather, they support an experience-based algorithm where flap choice is influenced by ulcer characteristics, patient mobility, and overall health. Future comparative studies are needed to validate the relative efficacy of these techniques and refine surgical decision-making further.

## Ethical approval

This study was approved by the Ethical Committee of Trung Vuong Hospital (REB approval number 1164). The research adhered to the principles outlined in the Declaration of Helsinki, ensuring the protection and confidentiality of participants throughout the study.

## Additional consent statement for patient photographs

Written informed consent was also obtained from all individual patients for whom identifying information and/or photographs are included in the article. The patients were informed about the publication of images and their explicit consent was secured.

## Author contributions

Tan Anh Tran: Conceptualization, methodology, data collection, manuscript writing. Khanh Trinh Quoc Pham: Conceptualization, methodology, data analysis, manuscript review and editing. Dat Ke Vo: Data collection, statistical analysis, manuscript review. Nhan Hoang Nguyen: Literature review, manuscript editing. Nhan Hong Nguyen: Literature review, manuscript editing. Phi Duong Nguyen: Supervision, data interpretation, manuscript editing.

## Data availability

Data are available upon reasonable request to the corresponding authors.

## Funding

No funding was received for this study.

## Conflict of interest

The authors declare that they have no known competing financial interests or personal relationships that could have appeared to influence the work reported in this paper.
